# Fluid Mechanics of Droplet Spreading of Chitosan/PVA-Based Spray Coating Solution on Banana Peels with Different Wettability

**DOI:** 10.3390/polym15214277

**Published:** 2023-10-31

**Authors:** Endarto Yudo Wardhono, Nufus Kanani, Mekro Permana Pinem, Dwinanto Sukamto, Yenny Meliana, Khashayar Saleh, Erwann Guénin

**Affiliations:** 1 Chemical Engineering, University of Sultan Ageng Tirtayasa, Cilegon 42435, Indonesia; nufus.kanani@untirta.ac.id; 2 Mechanical Engineering, University of Sultan Ageng Tirtayasa, Cilegon 42435, Indonesia; mekro_pinem@untirta.ac.id (M.P.P.); dwinanto@untirta.ac.id (D.S.); 3 Research Center for Chemistry, National Research and Innovation Agency, BRIN, Kawasan Puspiptek, Serpong, South Tangerang 15314, Banten, Indonesia; yenn005@brin.go.id; 4 Université de Technologie de Compiègne, ESCOM, TIMR (Integrated Transformations of Renewable Matter), Centre de Recherche Royallieu, CS 60 319, 60 203 Compiègne CEDEX, France; khashayar.saleh@utc.fr (K.S.); erwann.guenin@utc.fr (E.G.)

**Keywords:** CS/PVA solution, banana peels, spreading behavior, β_max_, scaling law analysis

## Abstract

The spreading behavior of a coating solution is an important factor in determining the effectiveness of spraying applications. It determines how evenly the droplets spread on the substrate surface and how quickly they form a uniform film. Fluid mechanics principles govern it, including surface tension, viscosity, and the interaction between the liquid and the solid surface. In our previous work, chitosan (CS) film properties were successfully modified by blending with polyvinyl alcohol (PVA). It was shown that the mechanical strength of the composite film was significantly improved compared to the virgin CS. Here we propose to study the spreading behavior of CS/PVA solution on fresh bananas. The events upon droplet impact were captured using a high-speed camera, allowing the identification of outcomes as a function of velocity at different surface wettabilities (wetting and non-wetting) on the banana peels. The mathematical model to predict the maximum spreading factor, β_max_, was governed by scaling law analysis using fitting experimental data to identify patterns, trends, and relationships between β_max_ and the independent variables, Weber (We) numbers, and Reynolds (Re) numbers. The results indicate that liquid viscosity and surface properties affect the droplet’s impact and spreading behavior. The Ohnesorge (Oh) numbers significantly influenced the spreading dynamics, while the banana’s surface wettability minimally influenced spreading. The prediction model reasonably agrees with all the data in the literature since the R^2^ = 0.958 is a powerful goodness-of-fit indicator for predicting the spreading factor. It scaled with $\beta_{max}=a+0.04{\left(We.Re\right)}^{1/3}$, where the “a” constants depend on Oh numbers.

## 1. Introduction

In recent years, developing bio films and coatings that protect fresh foods while maintaining their quality has been a crucial area of research and innovation. In this regard, bio-based polymers are an excellent solution to the challenges posed by synthetic polymers [1], and they can be derived from renewable resources such as plant-based feed stocks, agricultural waste, or algae [2]. Chitosan (CS) is a versatile biopolymer [3,4] that can be potentially applied as a preservative coating because it has an excellent film-forming ability [5]. Nevertheless, poor mechanical and gas barrier properties restrict its potential for widespread use. Blending CS with biodegradable synthetic polymers is one method to modify its characteristics and enhance flexibility [6]. To fully understand and harness its potential as a coating material, delving into the fluid mechanics and rheological properties of the chitosan-based solution is important.

Fluid mechanics plays a vital role in food engineering, especially when understanding or manipulating the characteristics of liquid droplets in processes involving spraying for coating applications [6]. Spray technology is widely used for various purposes, such as coating foods with flavorings, colorings, preservatives, or protective films. It allows for a controlled distribution of substances onto the food surface and provides a desired functional layer [7]. A spray is a dynamic collection of liquid droplets dispersed in a gas medium, usually created by fragmenting bulk liquid into smaller droplets [8]. Different devices can produce the spray, such as pressure nozzles, ultrasonic atomizers, or air across a liquid’s surface. The resulting droplet size and spray pattern can impact process efficiency, which is controlled by the microscopic properties of a single droplet [9]. Spreading, rebounding, splashing, and penetration are physical phenomena that might occur as a droplet of liquid impinges on a solid surface, depending on the fluid properties, impact conditions, wettability, and roughness of a surface [10]. The Weber (We) number We (inertia or surface tension forces), Ohnesorge (Oh) number (viscous forces and surface tension forces), and Reynolds (Re) number Re (inertia or viscous forces) are dimensionless parameters used to quantify different aspects of these phenomena [11].

The efficiency of the coating, particularly in terms of film thickness and barrier qualities, is greatly influenced by the spreading behavior of the liquid coating, which determines how evenly it spreads on the substrate surface and how quickly it forms a uniform film. In fresh food products, cuticle and epicuticular waxes act as a substantial barrier to wetting on the solid surface, causing droplets to bead up, bounce, or partially splash rather than spreading out and wetting the surface [12]. These may reduce the efficacy of the coating and render spray applications ineffective. Thus, controlling the surface characteristics to get uniform and continuous coating is important since it affects the surface’s ability to repel or absorb liquid. Several forces come into play, in which the competition between spreading and viscous forces is crucial in determining droplet dynamics [13]. Spreading is driven by the force that arises from the droplet’s attempt to minimize its surface energy and causes the droplet to flatten and increase its contact area with the solid surface. On the other hand, viscous forces oppose the spreading process, which tends to maintain the droplet’s structure and resist deformation [14]. If the spreading force is strong enough, the droplet will spread out, creating a thin film on the solid surface. The larger viscous forces might prevent the droplet from spreading completely. In this case, the droplet may keep a more spherical form, with a restricted contact area with the surface. The balance between the spreading and viscous forces determines the droplet’s final form and behavior. By controlling these forces, the processes involving droplet deposition, wetting, and coating may be manipulated [15]. However, it is important to note that other factors, such as surface roughness, gravity, and external flows, can also influence droplet dynamics.

The idea behind this work is to investigate the spreading behavior of a CS/PVA solution on the surface coating of an organic substrate (lady finger bananas) with different wettability in order to compare the adherence of the formulation to the fruit if the surface is adequately washed. Bananas are a highly perishable fruit, and improper handling after harvest can result in rapid deterioration, loss of quality, and reduced shelf life. Postharvest management techniques like coating can enhance shelf life by creating a protective barrier around the product [16]. Spray coating of fresh food requires a combination of technical knowledge, careful planning, and adherence to the products that ensures they are coated effectively while maintaining their quality and safety. Upon impact, cuticle and epicuticular waxes act as a substantial barrier to wetting on fruit peels, causing droplets to bead up, bounce, or partially splash rather than spreading out and wetting the surface [12].

In our previous work, we studied CS/polyvinyl alcohol (PVA) composite films fabricated using the solution casting technique with enhanced properties [17]. Here, to study the fluid mechanics of liquid droplet impacts on two banana surfaces, the evolution of the spreading droplets was observed as a function of the impact velocity and the wettability of the banana peels. The surface’s wettability was controlled by washing or not washing the fruit with tap water. The velocity of the droplets was estimated by measuring the distance between the needle tip and the peels. The viscosity and surface tension effects were determined by comparing the liquid droplet properties of the CS/PVA coating solution and water as a reference. A mathematical model is proposed by fitting experimental data regarding the spreading factor, which involves finding a statistical relationship that describes the data accurately.

## 2. Materials and Methods

### 2.1. Plant Substrates

Lady finger bananas were collected from a local market (Cilegon, Indonesia) and kept in a refrigerator at 15 °C before use. Prior to testing, the fruits were left at room temperature and washed or not washed with running tap water. The samples were peeled and divided into two treatments: unwashed (non-wetting) and washed (wetting surface). The peels were subsequently trimmed into a 4 cm × 4 cm rectangle and adhered to a nine cm diameter Petri dish.

### 2.2. Liquid Droplets

The coating solution for liquid droplets consisted of CS/PVA blends and deionized water (referred to as water). The methodology for preparing the CS/PVA solution has been described in our prior research [17], in which CS solution (1% *w/v* in 0.1 M of acetic acid) was mixed with PVA at the optimum ratio of CS/PVA (75/25) together with glycerol 10% *w*/*w* and succinic acid 5% *w*/*w* in a dry-basis CS/PVA blend.

### 2.3. Impact Measurement

**Figure 1:**
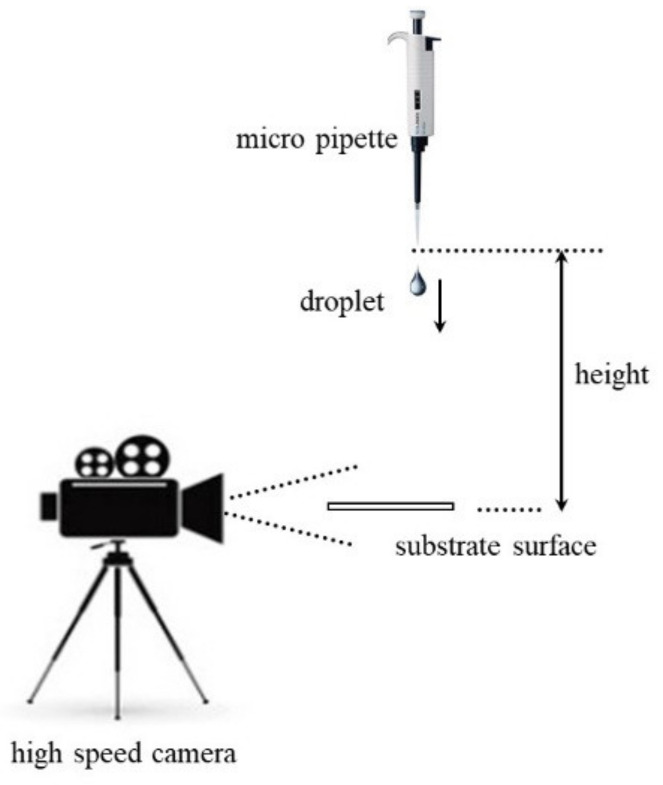
Experimental setup of impact measurement.

The experimental setup of the impact measurement is described in detail in our prior work [18], and the outline sketch of the experiment is shown in a diagram in Figure 1.

Experiments were conducted at various vertical velocities. A droplet was generated by pushing liquid through a micropipette, and it was released from a predetermined vertical height, in which the velocity of the droplet (v_o_) was determined by measuring the distance (h) between the tip and the substrate surface, $v_{0}=\sqrt{2gh}$. The droplet-impacting process was recorded using a high-speed camera. The acquisition rate was set to 2000 frames per second (fps), and the shutter speed was adjusted to 1/2000 s.

### 2.4. Impact Measurement

Liquid density at room temperature was measured using the pycnometric method using American Society for Testing and Materials (ASTM D854) [19]. Surface tension of the samples was evaluated at room temperature using a Kruss tensiometer and a Wilhelmy plate (KRUSS GmbH, Hamburg, Germany). The viscosity measurements of the liquid solution were conducted at 25 °C with a Physica MCR 301 rheometer (Anton Par GmbH, Graz, Austria), using concentric cylinder measuring system according to DIN 53019. The surface structure of the banana peel was observed using a VHX-5000 digital microscope (Olympus, Tokyo, Japan). The wettability of the organic substrate was evaluated via the contact angle measurement using a Drop shape Analyzer (DSA 100; KRUSS GmbH, Hamburg, Germany). ImageJ software NIH or equivalent was used to measure the droplet data, including initial diameter (D_0_), droplet spreading diameter D(t), maximum diameter (D_max_), and droplet height (h_D_).

## 3. Results and Discussion

### 3.1. Substrate Properties

**Figure 2:**
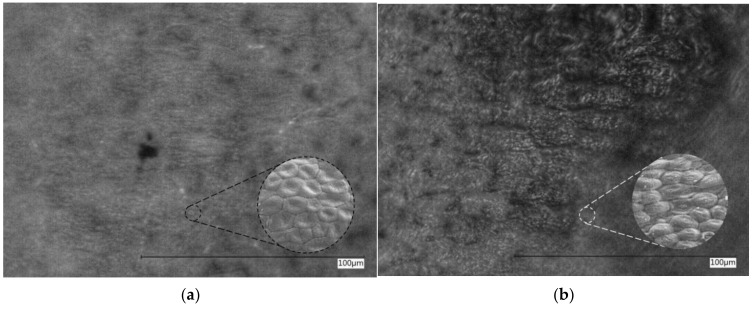
Surface morphology (2000× magnification) of the banana peels: (**a**) unwashed (non-wetting); (**b**) washed (wetting) surfaces.

The surface properties of the substrates were characterized using image processing techniques to observe the roughness and irregularities on a surface, while the wettability was determined by measuring mean contact angles and calculating Gibbs Adsorption Energy. A visual comparison of the two banana peels is illustrated in Figure 2.

The images reveal that the unwashed texture of the banana’s peel is smoother than the washed one. It exhibits a thin layer covering the surface, and some fractures and wrinkled structures were observed in the fruit that had been washed. The sessile drop method was used to determine the mean contact angle (CA), and the measurements were carried out three times for each sample to ensure reproducibility. The Gibbs adsorption energy (ΔG_ads_) was calculated using the contact angle data and the Young–Laplace equation [20]. The results are presented in Table 1.

Liquid water and CS/PVA demonstrate wetting behavior on the washed banana peel. CS/PVA exhibits better wettability with CA~63.8° than water CA~81.4°. Furthermore, both liquids display non-wetting characteristics on unwashed peel, where water has a higher contact angle with CA~108.1° than CS/PVA CA~98.4°. The same results are shown with ΔG_ads_ calculation, representing a thermodynamic quantity of molecule adsorption on the solid surface. Water has higher energies on both surfaces, −0.21 and 0.44 kJ/mole, compared to CS/PVA, which is −0.64 and 0.23 kJ/mole, respectively, for the washed and unwashed surfaces. A higher ΔG_ads_ might decrease the contact angle, suggesting that the liquid is better at wetting the surface due to stronger interactions with the surface molecules. On the other hand, for some systems, a higher ΔG_ads_ might increase the contact angle, indicating reduced wettability due to a higher degree of surface coverage by the adsorbed molecules onto a solid [21].

### 3.2. Droplet Properties and Impact Conditions

Physicochemical properties (density, *ρ*; viscosity, μ; and surface tension, σ) and the impact conditions (diameter, D_0_ and height, h) are listed in Table 2. Using water as the reference, the density of both liquids is quite similar, and the surface tension of CS/PVA is lower than water. The apparent viscosity of CS/PVA solution is approximately 12 times higher than water, which exhibits a shear-thinning (pseudo-plastic) flow behavior.

**Figure 3:**
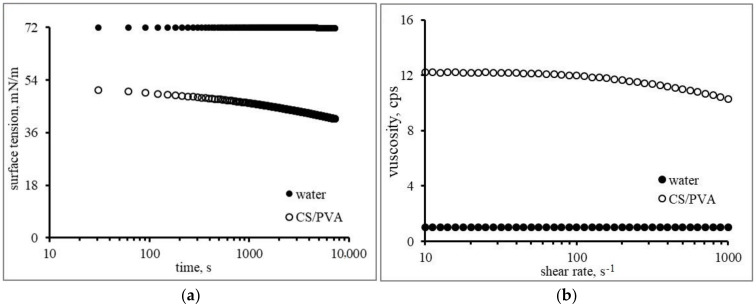
Liquid properties: (**a**) surface tension; (**b**) and viscosity at room temperature.

It is characteristic of a non-Newtonian fluid for the viscosity to decrease with increased shear rate. At the same time, the water has a constant viscosity independent of the applied shear rate characteristic of a Newtonian fluid (see Figure 3a,b).

For the impact parameters, the mean initial droplet diameter is D_0_ = 3.00 mm for liquid water and D_0_ = 2.85 mm for CS/PVA solution. Experiments were conducted at various heights ranging from 5 to 45 cm, with an impact velocity of 1 < *v*_o_ < 3 m/s. The impact dynamics characteristics acquired are Oh = 0.002 (40 < We < 400) for water and Oh = 0.030 (60 < We < 600) for CS/PVA. Both liquid droplets show a low Oh number (Oh < 1), indicating that viscous forces dominate, and surface tension effects are less significant [22]. The maximum We number for all tests is 600, which means that the impact is sufficiently low not to induce splashing [23].

### 3.3. Spreading Behavior

#### 3.3.1. Spreading on the Wetting Surface

**Figure 4:**
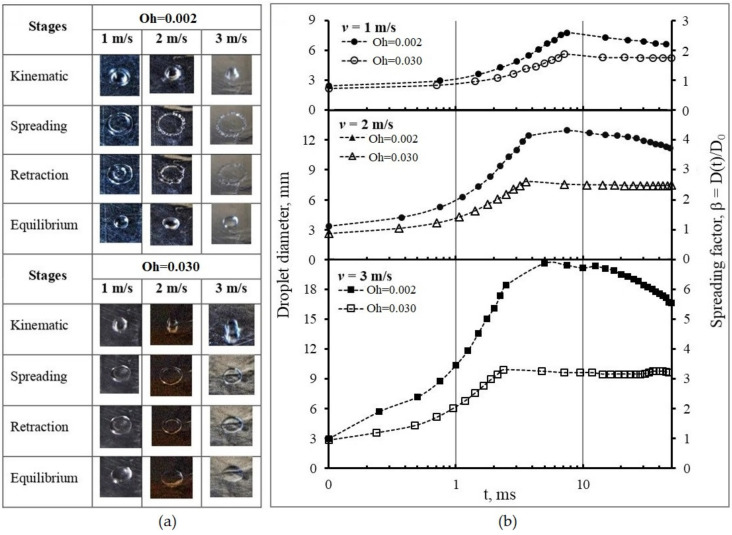
Evolution of droplet diameter at different impact velocities on the wetting surface: (**a**) images of spreading stages; (**b**) spreading pattern.

The time series of droplet impacts and subsequent spreading stages on the wetting surface for both liquids with Oh = 0.002 and Oh = 0.030 are shown in Figure 4a.

The impact characteristics at 1, 2, and 3 m/s were recorded by taking snapshots with the high-speed camera. Theoretically, there are four stages of the spreading following impact: kinematic, spreading, retraction, and equilibrium [24]. In general, all droplets are spherical in shape during the kinematic stage, represented by β < 1 [25]. Upon impact, the shape changes, resulting in a sudden halt in its vertical motion, causing the kinetic energy to be distributed across the liquid. The droplet continues to spread in the next stage. It spreads radially across the solid surface, driven by the remaining kinetic energy. The droplet shape becomes flatter, and its contact diameter on the surface increases and reaches maximum spreading (1 < β < β_max_) [26]. As the maximum value is attained, the droplet cannot spread further. The droplets show a lamella, or a pancake form, surrounded by a periphery. Surface tension tries to minimize the surface area of the liquid droplet in the retraction stage, causing it to recoil, leading to a continuous reduction in droplet diameter and its movement back to the impact point [27]. Deposition occurs during this stage. The droplets stay in this form and reach an equilibrium stage. Figure 4b shows the spreading pattern for two substances at 1, 2, and 3 m/s. It is observed that the droplet with Oh = 0.002 spreads faster than the droplet with Oh = 0.030 at all three impact speeds. The spreading behavior was significantly altered by the impact velocity, whereby D_max_ increased with *v*_0_, and the rate of receding decreased. The droplet with Oh = 0.002 reached its maximum value later than the one with Oh = 0.030, and these results are summarized in Table 3.

The droplets with Oh = 0.030 have smaller D_max_ of 5.01, 7.81, and 9.89 mm than the droplets with Oh = 0.002, in which D_max_ are 7.80, 12.96, and 20.61 mm at each impact speed. It can be attributed to the higher viscosity of the liquid with Oh = 0.03. As a shear-thickening fluid, the liquid acts almost like a solid when subject to rapid deformation. In this case, kinetic energy dissipates quickly upon impact, almost instantaneously converting the energy into heat or becoming stored as potential energy within the fluid structure. Internal friction limits the spread of the droplet. On the other hand, the water droplet with lower viscosity will spread out more upon impact due to a lower energy dissipation rate [28].

#### 3.3.2. Spreading on the Non-Wetting Surface

**Figure 5:**
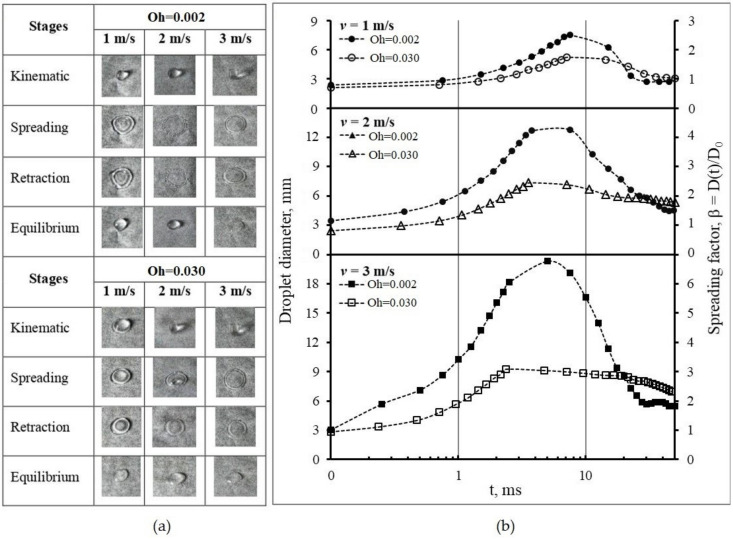
Evolution of droplet diameter at different impact velocities on the non-wetting surface: (**a**) images of spreading stages; (**b**) spreading pattern.

A visual observation of the spreading from both droplets on the non-wetting surface is presented in Figure 5a.

The captured images are at three impact speeds of 1, 2, and 3 m/s. All liquid droplets remain spherical at the kinematic stage. They rapidly create a thin film at the lower side while the upper side deforms into a semi-spherical form at the surface interface and no spreading lamella has been yet formed. Following the impact, the droplets expand radially, forming a flat film surrounded by a thick edge with maximum spreading. Subsequently, the edge undergoes contraction and thickening, ultimately merging with its inner boundary. The droplet with the lower Oh = 0.002 backs up to form a rounded shape, while the droplet with the higher Oh = 0.030 stays as a flat liquid layer. The droplets finally reach the equilibrium shape, with a diameter smaller than the D_max_. Figure 5b displays the spreading pattern of liquid droplets at 1, 2, and 3 m/s speed impacts. The maximum value of the droplet spreading during the spreading stage is summarized in Table 4. The figure shows that the droplets spread, as a parabolic curve, up to 50 ms. For the droplets with the lower Oh number = 0.002, the β_max_ obtained are 2.51, 4.27, and 6.78 for each speed. With the increase of speed, the β becomes steeper, indicating faster spreading and higher contact line velocity, whereas until maximum spreading, the β_max_ for the droplets with the higher Oh = 0.030 are 1.82, 2.58, and 3.25, which tend to decrease constantly for all speed variations.

The spreading phenomena of liquid droplets on the non-wetting banana show the same characteristics as on the wetting surface. The velocity affects the spreading in the non-wetting surface, in which *v*_0_ improved D_max_ and decreased the rate of receding significantly. The spreading times of the liquid droplets with Oh number = 0.030 are shorter (7.13, 3.56, and 2.38 ms) than the ones with Oh number = 0.002 (7.5, 7.5, and 5.0 ms) for each impact velocity. Shear-thinning non-Newtonian fluids of CS/PVA exhibit lower viscosity, leading to faster spreading on unwashed surfaces.

### 3.4. Maximum Spreading Factor

**Figure 6:**
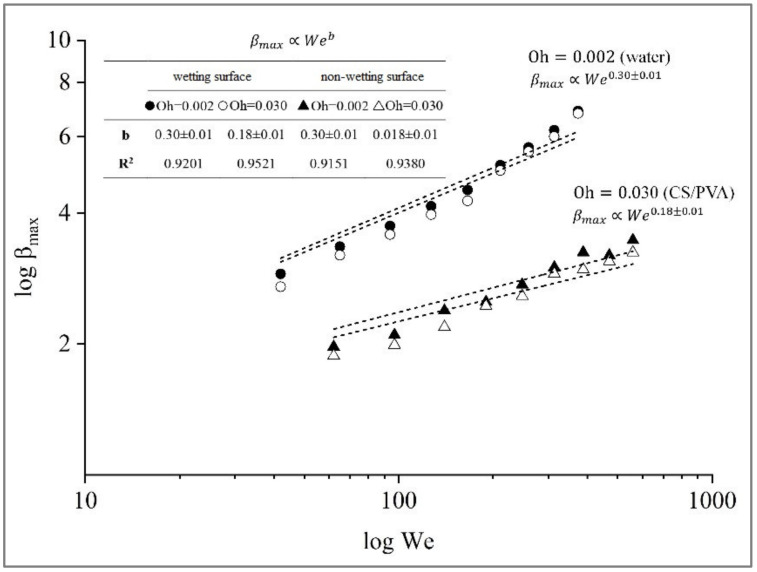
Maximum spreading factor, β_max_, as a function of We number on different surfaces.

The maximum spreading factor, β_max_, is a parameter used in the study of spreading phenomena, which represents the ratio of the largest lamella diameter, D_max_, over the initial one, D_0_. It quantifies how much a liquid droplet spreads out when it impacts a solid surface, which generally implies better surface coverage efficiency and therefore decrease in material consumption and reduction of waste. In this work, the β_max_ in a particular range of We numbers, (40 < We < 400) for Oh = 0.002 and (60 < We < 600) for Oh = 0.030, on both hydrophilic and hydrophobic surfaces is presented in Figure 6. The β_max_ is represented as a logarithmic function of We.

For all test cases, β_max_ values are distributed as a straight-line pattern with increasing We, suggesting that inertial forces become more dominant than surface tension. Inertial forces represent the kinetic energy associated with the liquid’s motion. On the other hand, surface tension is related to the cohesive forces at the liquid’s interface. It is in accordance with previous studies showing that inertia regulates how a material spreads onto a surface [26,29]. A higher Weber number signifies increased kinetic energy, resulting in a more significant perturbation of the droplet, leading to more energetic spreading behavior [30]. A lower Oh = 0.002 shows a higher β_max_. Increased viscosity leads to greater viscous friction forces in the near-wall boundary of the liquid layer. These prevent its spreading over the particle surface [31]. Two different banana surfaces have been examined, and the β_max_ data for these surfaces are nearly identical or very similar, showing that wettability minimally influences maximum spreading.

The prediction model obtained results that were in good agreement with the experimental results $\beta_{max}\propto {We}^{b}$ proposed by Clanet et al. [32], which predicted the maximum spreading for impacts on super-hydrophobic materials with a static contact angle over 150° by optimizing both surfaces, namely $\beta_{max}\propto {We}^{0.30\pm 0.01}$ for Oh = 0.002 and $\beta_{max}\propto {We}^{0.18\pm 0.01}$ for Oh = 0.030. To clarify the difference between the two exponents, the relationship β_max_ is dependent on the surface tension and viscosity characteristics.

### 3.5. Mathematical Model of Spreading

**Figure 7:**
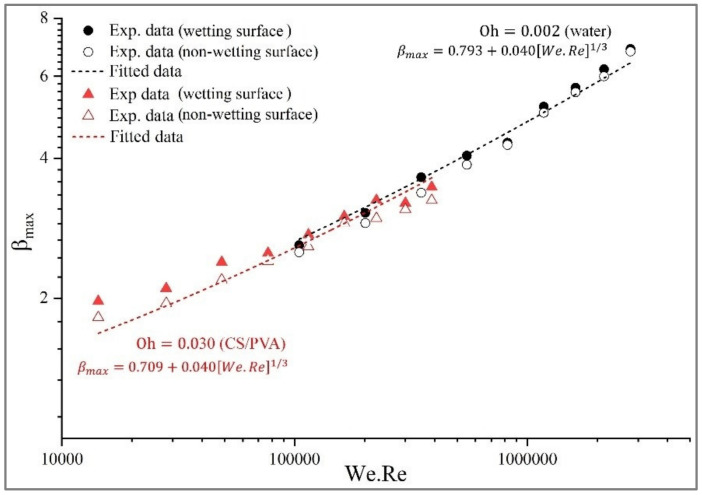
Reliability of model prediction for β_max_ on different wettabilities of the banana surface.

Generally, the three primary methods for predicting the β_max_ are scaling law analysis, the energy balance approach, and numerical simulation. These models are suitable for understanding the underlying physics phenomena in the spray process. They help to design process parameters to achieve desired coating thickness, distribution, and coverage. The scaling law describes how specific properties or behaviors change as a function of size or scale. It can be classified into two main categories based on the variables they use to express β_max_: (1) Allometric Scaling Models, to express β_max_ as a power-law function of size or scale of the system, such as We, Re, and θ, where the variable θ represents either the equilibrium contact angle or the advancing contact angle, with the latter having the potential to be dynamic or static, (2) and Isometric Scaling Models, to express β_max_ as a linear function of size, without any power-law exponent [33]. Several empirical investigations have been conducted to elucidate the dynamics of spreading phenomena [11,13,34,35,36,37]. Different models have been proposed for the prediction of β_max_. Scheller et al. [38] proposed an equation considering the correlation between the maximum spreading diameter with both Re and Oh that included two empirical coefficients, A and α. Tang et al. [26] conducted empirical investigations to determine various coefficients for five distinct surface values using the same parameters of scaling law. A similar method was utilized by Sen et al. [39] to empirically simulate the β_max_ of biofuel droplets on a stainless steel substrate. At the same time, Roisman et al. [40] proposed a semi-empirical equation that approximates the Navier–Stokes equations. This study presents a mathematical model for predicting the βmax at different Oh numbers on an organic surface using scaling law analysis. The model developed is based on the correlations found in experimental data. Figure 7 shows a non-linear regression model to fit the data into the power-law correlation. The obtained models are well fitted using correlating experimental results as a function of We and Re numbers. The fitting parameters and statistical factors, R-squared (R^2^) at different Oh numbers, are indicated in Table 5.

**Figure 8:**
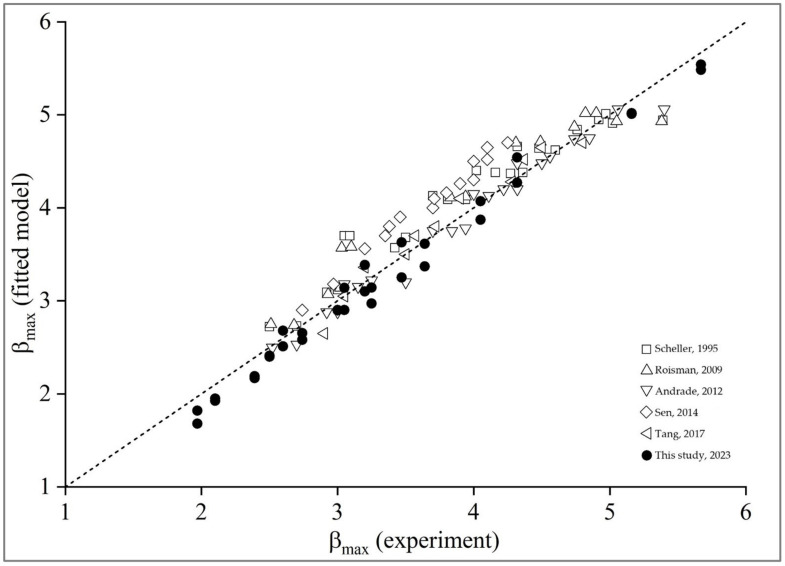
Comparison of model predictions and experimental data of β_max_ [26,38,39,40,41].

A comparison between previous published β_max_ data on difference surfaces and our empirical model is shown in Figure 8. The data were collected from Scheller et al. (1995) [38], Roisman et al. (2009) [40], Andrade et al. (2012) [41], Sen et al. (2014) [39], and Tang et al. (2017) [26]. The statistical values of each model are displayed in Table 6.

The best-fit model found by fitting experimental data is suggested:

For lower Oh (0.002):

(1) $$\beta_{max}=0.793+0.04{\left(We.Re\right)}^{1/3}$$

For higher Oh (0.030):

(2) $$\beta_{max}=0.709+0.04{\left(We.Re\right)}^{1/3}$$

The prediction model reasonably agrees with all the data in the literature. R-squared is greater than 0.958, a powerful goodness-of-fit indicator for predicting the maximum spreading factor. It scaled with $\beta_{max}=a+0.04{\left(We.Re\right)}^{1/3}$, where the constants “a” depend on Oh numbers.

## 4. Conclusions

This work studied the droplet spreading behavior of liquid CS/PVA blends and water as a reference on fresh banana surfaces with different surface wettabilities. The Oh number of liquid droplets (0.002 for water and 0.030 for CS/PVA) is less than 1, indicating that viscous forces dominate, and surface tension effects are less significant. The We number for all tests is up to 600, meaning that the impact is sufficiently low not to allow splashing. The liquid viscosity and surface properties affect the droplets’ impact behavior. Upon impact, water and CS/PVA droplets spread radially outwards on wetting surfaces, form a lamella surrounded by a periphery, and reach a maximum diameter. The surface tension causes the droplets to recoil, reduce the diameter, return to the impact point, stay in this form, and reach equilibrium. On the non-wetting surface, both liquids expand as a flat film surrounded by a thick edge and get the maximum spreading. Subsequently, the edge undergoes contraction and thickening, ultimately merging with its inner boundary. The water droplet backs up to form a rounded shape, while CS/PVA stays as a flat liquid layer. The droplets finally reach an equilibrium shape with a smaller diameter than the D_max_. The spreading factor β, which is a function of impact velocity, demonstrates the primary role of surface tension and viscosity. The Oh numbers significantly influence the spreading dynamics. The β_max_ data for two different banana surfaces are nearly identical or very similar, indicating the banana’s surface wettability minimally influences the maximum spreading. The prediction model reasonably agrees with all the data from the literature, since R^2^ = 0.958 is a powerful goodness-of-fit indicator for predicting the maximum spreading factor. It scaled with $\beta_{max}=a+0.04{\left(We.Re\right)}^{1/3}$, where the constants “a” depend on Oh numbers.

## Figures and Tables

**Table 1:** Wettability determination on the banana surface for the two liquids.

Liquid	Banana Peels			
	Washed Surface		Unwashed Surface	
	CA (°)	ΔG (kJ/mole)	CA (°)	ΔG (kJ/mole)
Water	81.4 ± 1.8	−0.21	108.1 ± 2.1	0.44
CS/PVA	63.8 ± 2.5	−0.64	98.4 ± 2.4	0.23

**Table 2:** Liquid properties at room temperature and droplet impact conditions.

Liquids	*ρ*	μ	σ	D_o_	h	$Oh=\frac{\mu }{\sqrt{\rho vD_{0}}}$	$W_{e}=\frac{\rho D_{0}v_{0}^{2}}{\sigma }$
	g/cm^3^	cps	mN/m	mm	cm		
Water	0.998	1.00	72.00	3.00 ± 0.05	5–45	0.002	40–400
CS/PVA	1.125	12.25	51.62	2.85 ± 0.05	5–45	0.030	60–600

**Table 3:** Maximum value of droplet spreading on the wetting surface.

Oh	*v* = 1 m/s			*v* = 2 m/s			*v* = 3 m/s		
	t	D_max_	β_max_	t	D_max_	β_max_	t	D_max_	β_max_
	ms	mm	-	ms	Mm	-	ms	mm	-
0.002	7.50	7.80	2.60	7.50	12.96	4.32	5.00	20.61	6.87
0.030	7.13	5.01	1.97	3.56	7.81	2.74	2.38	9.89	3.47

**Table 4:** Maximum value of droplet spreading at hydrophilic surface.

Oh	*v* = 1 m/s			*v* = 2 m/s			*v* = 3 m/s		
	t	D_max_	β_max_	t	D_max_	β_max_	t	D_max_	β_max_
	ms	mm	-	ms	mm	-	Ms	mm	-
0.002	7.50	7.53	2.51	7.50	12.81	4.27	5.00	20.34	6.78
0.030	7.13	5.19	1.82	3.56	7.35	2.58	2.38	9.26	3.25

**Table 5:** Fitting parameters of the β_max_ on the solid surface.

Oh	Fitting Parameters			
	a	b	c	R^2^
0.002	0.793	0.040	1/3	0.9623
0.030	0.709	0.040	1/3	0.9539

**Table 6:** Statistical values of the different models.

Literature	Fitted Model	R^2^
Scheller, 1995 [38]	$\beta_{max}=0.61{\left({Re}^{2}Oh\right)}^{0.166}$	0.941
Roisman, 2009 [40]	$\beta_{max}=0.87{Re}^{0.2}-0.4\left({Re}^{0.4}/\sqrt{We}\right)$	0.933
Andrade, 2012 [41]	$\beta_{max}=1.28+0.071{We}^{0.25}{Re}^{0.25}$	0.972
Sen, 2014 [39]	$\beta_{max}=1.73{We}^{0.14}$	0.900
Tang, 2017 [26]	$\beta_{max}=a{\left(We/Oh\right)}^{b}$, a & b depend on roughness	0.966
This study, 2023	$\beta_{max}=a+0.04{\left(We.Re\right)}^{1/3}$, “a” depend on Oh	0.954
